# Assessment of left ventricular deformation in patients with type 2 diabetes mellitus by cardiac magnetic resonance tissue tracking

**DOI:** 10.1038/s41598-020-69977-x

**Published:** 2020-08-04

**Authors:** Lin-jun Xie, Zhi-hui Dong, Zhi-gang Yang, Ming-yan Deng, Yue Gao, Li Jiang, Bi-yue Hu, Xi Liu, Yan Ren, Chun-chao Xia, Zhen-lin Li, Hua-peng Zhang, Xiao-yue Zhou, Ying-kun Guo

**Affiliations:** 1 Department of Radiology, Key Laboratory of Obstetric & Gynecologic and Pediatric Diseases and Birth Defects of Ministry of Education, West China Second University Hospital, Sichuan University, 20# South Renmin Road, Chengdu, 610041 Sichuan China; 2 Department of Radiology, Luoyang Central Hospital Affiliated to Zhengzhou University, 288# Zhongzhou Middle Road, Luoyang, 471009 Henan China; 3 Department of Radiology, West China Hospital, Sichuan University, 37# Guo Xue Xiang, Chengdu, 610041 Sichuan China; 4 Department of Endocrinology and Metabolism, West China Hospital, Sichuan University, 37# Guo Xue Xiang, Chengdu, 610041 Sichuan China; 5 MR Collaboration, Siemens Healthineers Ltd., Shanghai, China

**Keywords:** Diabetes complications, Magnetic resonance imaging

## Abstract

To quantify the global and regional left ventricular (LV) myocardial strain in type 2 diabetes mellitus (T2DM) patients using cardiac magnetic resonance (CMR) tissue-tracking techniques and to determine the ability of myocardial strain parameters to assessment the LV deformation. Our study included 98 adult T2DM patients (preserved LV ejection fraction [LVEF], 72; reduced LVEF, 26) and 35 healthy controls. Conventional LV function, volume-time curve parameters and LV remodeling index were measured using CMR. Global and regional LV myocardial strain parameters were measured using CMR tissue tracking and compared between the different sub-groups. Receiver operating characteristic analysis was used to assess the diagnostic accuracy. Regression analyses were conducted to determine the relationship between strain parameters and the LV remodeling index. The results show that global radial peak strain (PS) and circumferential PS were not significantly different between the preserved-LVEF group and control group (*P* > 0.05). However, longitudinal PS was significantly lower in the preserved-LVEF group than in the control group (*P* = 0.005). Multivariate linear and logistic regression analyses showed that global longitudinal PS was independently associated (β = 0.385, *P* < 0.001) with the LV remodeling index. In conclusion, early quantitative evaluation of cardiac deformation can be successfully performed using CMR tissue tracking in T2DM patients. In addition, global longitudinal PS can complement LVEF in the assessment of cardiac function.

## Introduction

Type 2 diabetes mellitus (T2DM) is a metabolic disease associated with high morbidity. It is characterized by insufficient or non-effective utilization of insulin, resulting in chronic hyperglycemia^1^. T2DM is presently a rapidly growing global public health problem. According to the 2015 data of the International Diabetes Federation (IDF), more than 450 million people worldwide have DM^2^. Moreover, the number of T2DM patients is still increasing. The IDF predicts that the number of DM patients will reach 600 million by 2040^2^. Cardiovascular disorders have been reported to be the leading causes of death and disability in T2DM patients^3^.

T2DM patients have been found to have different types of cardiovascular issues, such as increased oxidative stress, endothelial dysfunction, mitochondrial dysfunction, impaired calcium handling, and extracellular matrix remodeling^4^. These findings suggest that histological changes, LV remodeling, myocardial hypertrophy, and fibrosis occur, which may lead to cardiac diastolic dysfunction and eventually to systolic heart failure^4^. Early studies have also suggested that T2DM might be associated with concentric LV remodeling which may lead to systolic or diastolic dysfunction^5,6^. Concentric LV remodeling itself has been reported to be an adverse prognostic marker of cardiovascular events, and such remodeling may explain why T2DM patients with heart failure have such a poor prognosis^7,8^. However, early myocardial damage is slow and difficult to detect in T2DM patients, and symptoms are not specific, leading to an increase in the risk of hospitalization and adverse outcomes^9,10^. Therefore, early evaluation of myocardial damage using imaging approaches is extremely important in making an early diagnosis, commencing treatment promptly, and hopefully preventing a poor prognosis in T2DM patients.

Clinically, left ventricular ejection fraction (LVEF) is widely used to evaluate cardiac function and thus myocardial status^11^. However, in the early stages of many cardiac diseases, LVEF is preserved despite impaired myocardial contractility^12,13^. Myocardial strain is an important method to evaluate the clinical or subclinical deformation of the cardiac^14^. Current methods for measuring myocardial strain include echocardiography, MR tagging and CMR tissue tracking. Echocardiography has been used extensively for strain analysis, but the accuracy of echocardiographic results depends substantially on operator skill and is limited by narrow acoustic windows. MR tagging require special sequences, and post-processing is complex. CMR has been regarded as the golden standard for the accurate quantification of cardiac function. In recent years, CMR tissue tracking has rapidly developed into a quantitative technique for the quick evaluation of myocardial strain^15^. CMR tissue tracking assesses the movement of the myocardial voxel and provides unique information about myocardial strain in subclinical conditions and potentially identifies myocardial damage before a significant reduction in LVEF occurs^16,17^. Traditional SSFP cine sequences (bFFE, TrueFISP, and FIESTA) are suitable for CMR tissue tracking and only require a relatively quick and easy post-processing approach making these sequences ideal for the quantitative evaluation of LV myocardial strain characteristics. Presently, few studies are focusing on the assessment of myocardial strain damage using CMR tissue tracking in T2DM patients^18^. Thus, in the present study, we aimed to quantify myocardial strain using CMR tissue tracking, in T2DM patients, especially in patients with preserved LVEF, and to determine the ability of myocardial strain parameters to assessment the LV deformation. We explore the relationship between myocardial strain parameters with LV remodeling index.

## Materials and methods

This study was approved by the Ethics Committee of Clinical Trials and Biomedicine at the West China Hospital of the Sichuan University (No-2016-24) and we pledged to abide by the declaration of Helsinki (2000 EDITION) in accordance with the relevant medical research rules in the study. Informed consent was obtained from all participants prior to study participation. All participant-sensitive information was kept confidential and was used solely for the purpose of this study.

### Study population

One hundred and three adult T2DM patients from West China Hospital of Sichuan University were enrolled in this study between March 2016 and May 2018. Inclusion criteria were diagnosis of T2DM in accordance with the criteria of the American Diabetes Association^19^. The exclusion criteria were history of cardiovascular disease (i.e. cardiomyopathy, congenital heart disease, pulmonary heart disease, myocardial infarction, valvular disease, arrhythmia, etc.), symptoms of possible cardiovascular disease (i.e. chest pain, palpitations, or dyspnea), malignant tumors, uncontrollable hypertension, thyroid disease, other systemic diseases, and contraindications to CMR. Eventually, 98 T2DM patients of the original 103 fulfilled these criteria and were assessed in the study. Exclusion criteria for the healthy controls were history of diabetes mellitus, hypertension, hyperlipidemia, cardiovascular diseases, malignant tumors, thyroid diseases, other systemic diseases, and contraindications of CMR. Concurrently, thirty-five individuals from our healthy volunteer database, with similar sex, age and body mass index [BMI] distribution to those of our patients, were recruited to constitute the control group.

### Basic information and laboratory data collection

For all T2DM patients, we recorded age, sex, disease duration, blood pressure, height, and weight; we then calculated BMI. Additionally, we collected the following laboratory data from each T2DM patient: HbA1c, triglyceride (TG) level, total cholesterol, high-density lipoprotein (HDL) cholesterol, and low-density lipoprotein cholesterol (LDL).

### CMR protocol

All patients and normal controls underwent CMR on the 3 T whole-body MR scanner MAGNETOM Skyra (Siemens Healthcare) with an 18-channel body phased-array coil combined with a spine coil (12 of 32 channels used). All participants were examined in the supine position and were required to hold their breaths at predefined portions of the exam. The manufacturer’s electrocardiographic (ECG) gating device was used during the entire examination. Following local transverse, coronal, and sagittal imaging, a series of short-axis cine images were acquired from the mitral valve level to the LV apex using the steady-state free-precession sequence with retrospective ECG gating (repetition time [TR], 39.34 ms; echo time [TE], 1.22 ms; flip angle, 38°; slice thickness, 8 mm; field of view, 340 × 285 mm2; matrix size, 208 × 166; and 25 frames per cardiac cycle). In addition, four-chamber and two-chamber long-axis cine images were acquired.

### Image analysis

All CMR data were analyzed using the commercially available software cvi^42^ (Circle Cardiovascular Imaging, Inc.). Image analysis was performed to evaluate conventional cardiac function and myocardial strain.

**Figure 1:**
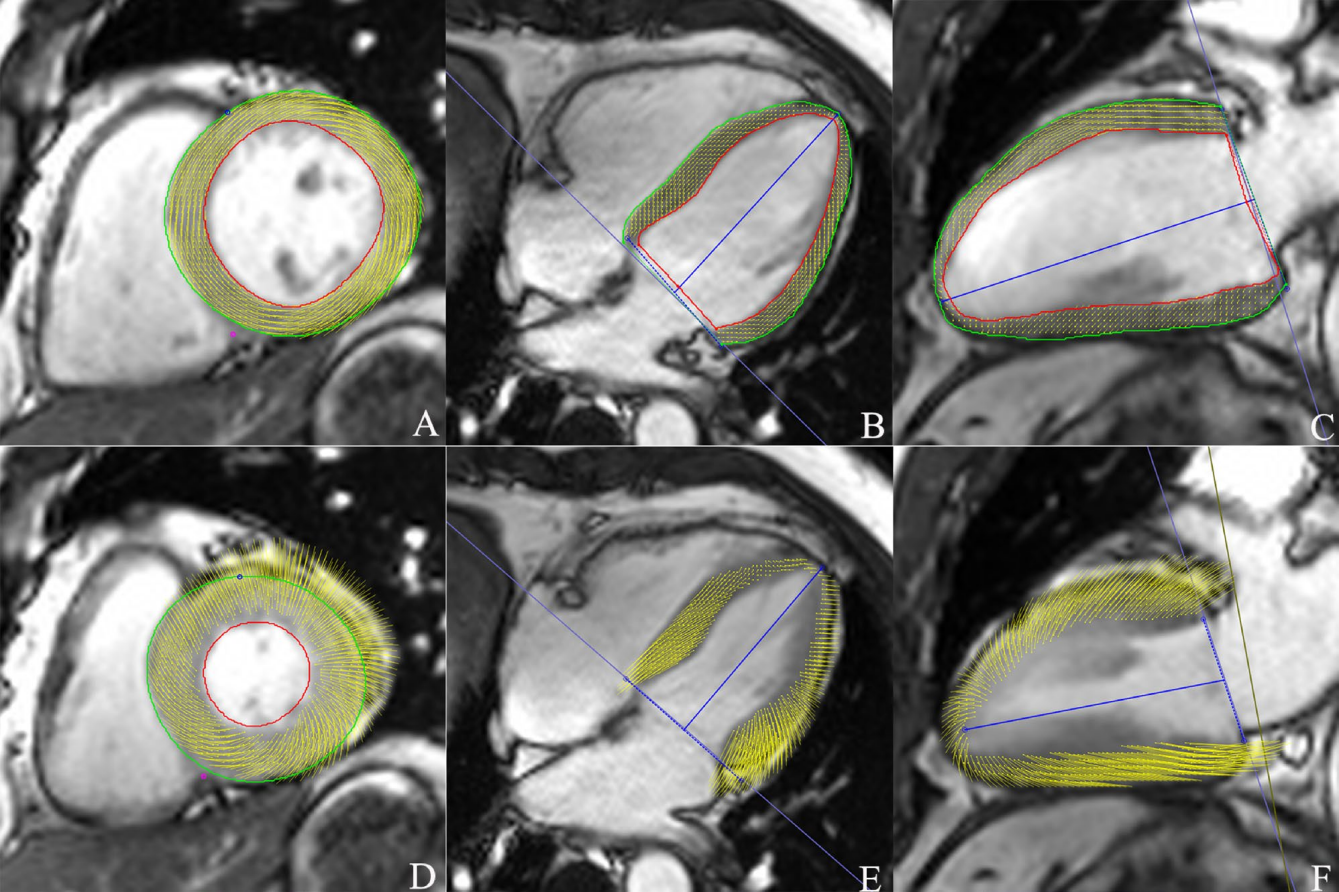
**A**–**F** Cardiac magnetic resonance tissue tracking in short-axis and long-axis two-chamber and four-chamber cine images at end-diastole (**A**–**C**) and end-systole (**D**–**F**). cvi^42^ (version 5.9.1; Circle Cardiovascular Imaging, Inc.)

A set of short-axis and long-axis two-chamber and four-chamber slices were uploaded into the tissue-tracking module. Only short-axis slices were used to analyze cardiac function while both short- and long-axis slices were used to analyze strain parameters. An experienced radiologist manually delineated the endocardial and epicardial borders in the LV end-diastolic and end-systolic phases for each series involving short-axis two-chamber and four-chamber cine images; the moderator bands and papillary muscles were carefully excluded. Finally, reference lines were marked in the LV long-axis two-chamber and four-chamber cine images, and the short-axis reference points were defined as shown in Fig. 1.

Short-axis cine images were used to analyze cardiac function with the cvi^42^ short-3D module. Cardiac function indexes, including LV end-diastolic volume (EDV), end-systolic volume (ESV), ejection fraction (EF), stroke volume (SV), cardiac output (CO), and cardiac mass, were then automatically computed. The LV end-diastolic dimension (LVEDD) was measured on a four-chamber cine image. The LV remodeling index was obtained by calculating the ratio of the LV mass to the LV EDV. cvi^42^ software was used to automatically calculate the time-volume curve parameters, including the peak ejection rate (PER) and peak filling rate (PFR).

The different strain parameters of each phase were automatically calculated by tracking the myocardial voxel points. The end-diastolic phase was the initial point of strain tracking. The LV global and regional (i.e. basal, mid, and apical segments) tissue-tracking variables, including the radial, circumferential, and longitudinal peak strain (PS), peak systolic strain rate (PSSR), and peak diastolic strain rate (PDSR), were automatically computed. Radial strain and circumferential strain represent movement in the cardiac short-axis direction. Radial strain reflects the thickening of the ventricular wall in the systolic phase, and circumferential strain is defined as circular motion in the direction of the short axis. Longitudinal strain refers to strain in the cardiac long-axis direction, which is the average strain of each longitudinal myocardial fiber segment. As LV wall thickening increases with wall contraction, radial strain is expressed as a positive value. Conversely, as the myocardium shortens in the longitudinal and circumferential directions during LV contraction, circumferential strain and longitudinal strain are expressed as negative values^20,21^.

### Reproducibility

The reproducibility of the global LV PS parameters was assessed by two experienced radiologists. To assess intra-observer variability, a single observer completed measurements of 24 random cases at two different time points with an interval of 1 month; these measurements from the two different time points were compared. To assess inter-observer variability, measurements from two independent experienced observers blinded to each other’s findings were compared.

### Statistical analysis

All data were analyzed using the Kolmogorov–Smirnov test. Normal data are presented as mean values with standard deviations. The homogeneity of variance assumption was assessed using Levene’s test. Comparisons were made regarding all cardiac function indexes and strain parameters between the reduced-LVEF group, the preserved-LVEF group, and the control group. Continuous variables were compared using the independent Student’s *t*-test or the one-way analysis of variance. The Mann–Whitney *U*-test was used to compare data not showing a normal distribution. Pearson’s correlation analyses were used to evaluate possible correlations of tissue-tracking variables with LVEF and time-volume curve parameters. Univariate and multivariate linear regression analyses were used to identify independent correlates of LV strain parameters and the LV remodeling index (LVMVR:LV mass to LV end diastolic volume). Multivariate logistic regression analyses were performed to explore the influence factor of LV remodeling. Receiver operating characteristic (ROC) analysis was performed to determine optimal cut-off values for LV strain parameters to identify LV dysfunction in T2DM patients. The intra-observer and inter-observer variabilities (reproducibility) were assessed using intraclass correlation coefficients (ICCs). All statistical tests were two-tailed. All statistical analyses were performed using SPSS (version 24.0; IBM Corp.) and GraphPad Prism (version 7.0; GraphPad Software). A *P*-value < 0.05 was considered to indicate a statistically significant difference.

## Results

### Baseline characteristics

Of the 98 T2DM patients, 72 had preserved LVEF (LVEF ≥ 55%; 38 men; mean age, 57.8 ± 9.7 years; preserved-LVEF group), and 26 had reduced LVEF (LVEF < 55%; 16 men; mean age, 55.5 ± 11.6 years; reduced-LVEF group). The 35 healthy controls consisted of 17 men with a mean age of 53.2 ± 10.3 years. The baseline characteristics of the T2DM patients and normal controls are presented in Table 1. There were no significant differences in laboratory data between the preserved-LVEF group and the reduced-LVEF group (all *P* > 0.05).

**Table 1:** Baseline Characteristics and CMR parameters of normal individuals, T2DM patients.

	Normal n = 35	Patients with preserved LVEF n = 72	Patients with reduced LVEF n = 26
**Baseline characteristics**			
Age, years	53.23 ± 10.28	57.82 ± 9.73	55.54 ± 11.55
Male, n (%)	17, 49%	38, 53%	16,62%
Duration (years)	–	7.59 ± 6.75	7.92 ± 6.61
BSA, m^2^	1.61 ± 0.11	1.63 ± 0.15	1.63 ± 0.17
BMI, kg/m^2^	23.56 ± 2.04	23.59 ± 2.53	23.94 ± 2.98
HR	73.69 ± 10.66	74.34 ± 12.05	74.48 ± 10.57
Systolic blood pressure (mmHg)	126.63 ± 7.13	130.71 ± 13.53	130.31 ± 16.22
Diastolic blood pressure (mmHg)	78.77 ± 7.60	80.69 ± 9.20	77.62 ± 13.08
HbA1c, %	5.27 ± 0.31	7.41 ± 2.19	7.52 ± 2.04
TG	1.24 ± 0.32	1.81 ± 1.86	1.84 ± 1.35
TC	4.42 ± 0.61	4.48 ± 1.22	4.69 ± 1.37
HDL	1.36 ± 0.27	1.31 ± 0.46	1.31 ± 0.56
LDL	2.68 ± 0.60	2.54 ± 0.86	2.59 ± 1.00
**CMR parameters**			
LVEDV (ml)	119.78 ± 21.56	115.56 ± 21.62	131.61 ± 41.80
LVESV (ml)	44.69 ± 10.12	43.00 ± 10.32	69.21 ± 29.01*^,§^
LVSV (ml)	75.08 ± 13.07	72.56 ± 13.14	62.40 ± 16.71*
CO (l/min)	5.43 ± 1.04	5.36 ± 1.17	4.62 ± 1.20*^,§^
LVEF, %	62.86 ± 3.63	62.98 ± 4.04	48.52 ± 6.21*^,§^
Mass (g)	76.29 ± 16.23	86.90 ± 22.46*	104.16 ± 34.54*
LVEDD (mm)	47.03 ± 3.12	46.36 ± 4.38	48.60 ± 5.19
LVMVR	0.65 ± 0.15	0.76 ± 0.16*	0.81 ± 0.22*
PER (ml/s)	367.78 ± 72.14	348.03 ± 75.15	300.01 ± 71.13*^,§^
PFR (ml/s)	326.81 ± 75.93	279.56 ± 63.24*	253.56 ± 63.36*

Data given as the mean ± SD.

*BSA* body surface area, *BMI* body mass index, *HR* heart rate, *TG* triglyceride, *TC* cholesterol, *HDL* high-density lipoprotein, *LDL* low density lipoprotein, *LVEDV* left ventricular end diastolic volume, *LVESV* left ventricular end systolic volume, *SV* stroke volume, *CO* cardiac output, *EF* ejection fraction, *LVEDD* left ventricular end-diastolic dimension, *PER* peak ejection rate, *PFR* peak filling rate.

*P < 0.05 versus normal group.

^§^P < 0.05 versus T2DM with preserved LVEF (LVEF ≥ 55%).

### Comparisons of the LV function and time-volume curve parameters

LVEF were lower in the T2DM patients as compared to the control group (59.14 ± 7.94% vs. 62.86 ± 3.63%, *P* < 0.001). The mass was significantly higher in the T2DM patients as compared to the control group (91.48 ± 27.12 g vs. 76.29 ± 16.23 g, *P* < 0.001).

Conventional LV function and time-volume curve parameters of subgroups were obtained and compared (Table 1). Among all the LV function indexes on CMR, LVESV, SV, CO, mass, and LVMVR were significantly higher in the reduced-LVEF group as compared to the control group (all *P* < 0.05). On the other hand, LVEDV, LVESV, SV, and CO were not significantly different between the control group and the preserved-LVEF group (*P* > 0.05). The time-volume curve parameter PER was significantly lower in the reduced-LVEF group as compared to the control group (*P* = 0.002). This index was numerically lower in the preserved-LVEF group as compared to the control group, but the difference was not significant (*P* = 0.586). PFR was significantly lower in both the reduced- and preserved-LVEF groups as compared to the control group (253.57 ± 63.36 ml/s vs. 326.81 ± 75.93 ml/s, *P* < 0.001 and 279.56 ± 63.24 ml/s vs. 326.81 ± 75.93 ml/s, *P* = 0.002, respectively).

### Global and regional myocardial strain indexes

The LV global radial, circumferential, and longitudinal PS were lower in the T2DM patients as compared to the control group (all *P* < 0.05). Although there were no significant differences between the T2DM patients and the control group in the LV global radial and circumferential PDSR (*P* > 0.05), global longitudinal PDSR was lower in the T2DM patients as compared to the control group (0.83 ± 0.21(1/S) vs. 0.93 ± 0.16(1/S), *P* < 0.05). There were no significant differences between the T2DM patients and the control group in the LV global radial, circumferential and longitudinal PSSR (*P* > 0.05).

**Figure 2:**
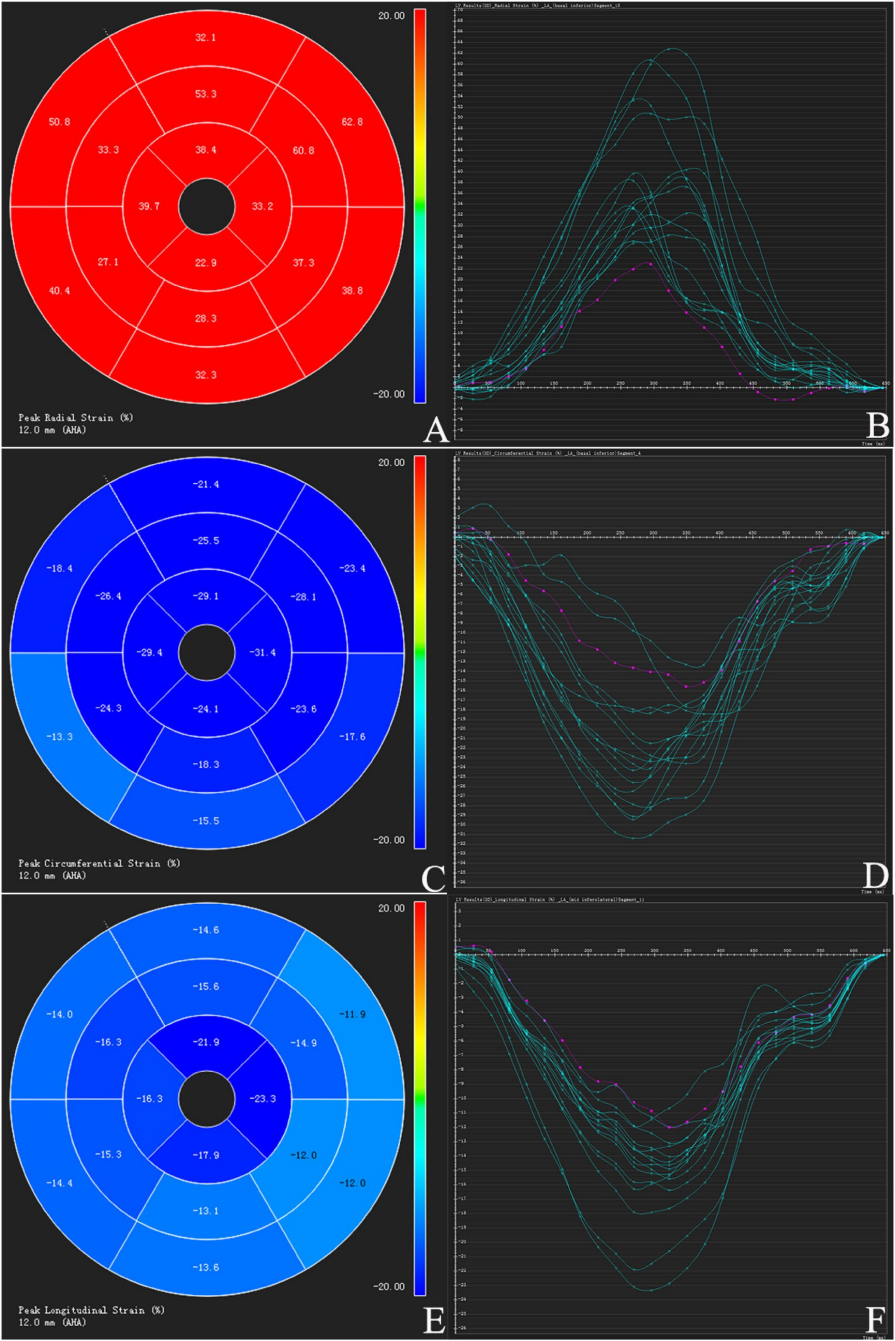
Tissue-tracking variables in a type 2 diabetes mellitus patient. (E/F) General reduction (coloration from dark blue to light blue) in longitudinal peak strain (absolute value). cvi^42^ (version 5.9.1; Circle Cardiovascular Imaging, Inc.)

Global strain parameters of subgroups are shown in Table 2. The LV global radial, circumferential, and longitudinal PS were lower in the reduced-LVEF group as compared to the control group (all *P* < 0.001). Although there were no significant differences between the preserved-LVEF group and the control group in the LV global radial and circumferential PS (*P* > 0.05), global longitudinal PS was lower in the preserved-LVEF group as compared to the control group (− 15.93 ± 3.12% vs. − 17.79 ± 2.02%, *P* = 0.005) (Fig. 2).

**Table 2:** Left ventricle global deformation difference between normal patients, preserved and reduced LVEF T2DM patients.

	Normal n = 35	Patients with preserved LVEF n = 72	Patients with reduced LVEF n = 26
Radial PS (%)	44.90 ± 8.07	44.43 ± 8.78	30.48 ± 7.87*^,§^
Circumferential PS (%)	− 20.40 ± 1.68	− 20.25 ± 2.26	– 16.18 ± 2.62*^,§^
Longitudinal PS (%)	− 17.79 ± 2.02	− 15.93 ± 3.12*	− 13.97 ± 2.64*^,§^
Radial PSSR (1/S)	2.83 ± 0.80	2.69 ± 0.98	1.89 ± 0.77*^,§^
Circumferential PSSR (1/S)	− 1.09 ± 0.19	− 1.07 ± 0.23	− 0.78 ± 0.40*^,§^
Longitudinal PSSR (1/S)	− 0.93 ± 0.16	− 0.85 ± 0.21	− 0.78 ± 0.19*
Radial PDSR (1/S)	− 3.45 ± 0.95	− 2.97 ± 0.92*	− 2.03 ± 0.75*^,§^
Circumferential PDSR (1/S)	1.29 ± 0.28	1.14 ± 0.25*	0.99 ± 0.25*^,§^
Longitudinal PDSR (1/S)	1.11 ± 0.23	0.89 ± 0.30*	0.84 ± 0.22*

*PS* peak strain, *PSSR* peak systolic strain rate, *PDSR* peak diastolic strain rate.

*P < 0.05 versus control group.

^§^*P* < 0.05 versus T2DM with preserved LVEF.

With regard to the global systolic strain indexes, the global radial, circumferential, and longitudinal PSSR were significantly lower in the reduced-LVEF group than in the control group (radial: 1.89 ± 0.77 1/s vs. 2.83 ± 0.80 1/s, *P* < 0.001; circumferential: − 0.78 ± 0.40 1/s vs. − 1.09 ± 0.19 1/s, *P* < 0.001; longitudinal:  − 0.78 ± 0.19 1/s vs. − 0.93 ± 0.16 1/s, *P* = 0.012). However, the global systolic strain indexes were not significantly different between the control group and the preserved-LVEF group (*P* > 0.05). With regard to the global diastolic strain indexes, the global radial, circumferential, and longitudinal PDSR were significantly lower in both the preserved- and reduced-LVEF groups than in the control group (radial: − 2.97 ± 0.92 1/s vs. − 3.45 ± 0.95 1/s, *P* = 0.031 and − 2.03 ± 0.75 1/s vs. − 3.45 ± 0.95 1/s, *P* < 0.001; circumferential: 1.14 ± 0.25 1/s vs. 1.29 ± 0.28 1/s, *P* = 0.012 and 0.99 ± 0.25 1/s vs. 1.29 ± 0.28 1/s, *P* < 0.001; longitudinal: 0.89 ± 0.30 1/s vs. 1.11 ± 0.23 1/s, *P* = 0.001 and 0.84 ± 0.22 1/s vs. 1.11 ± 0.23 1/s, *P* = 0.001, respectively) (Supplementary Information).

**Figure 3:**
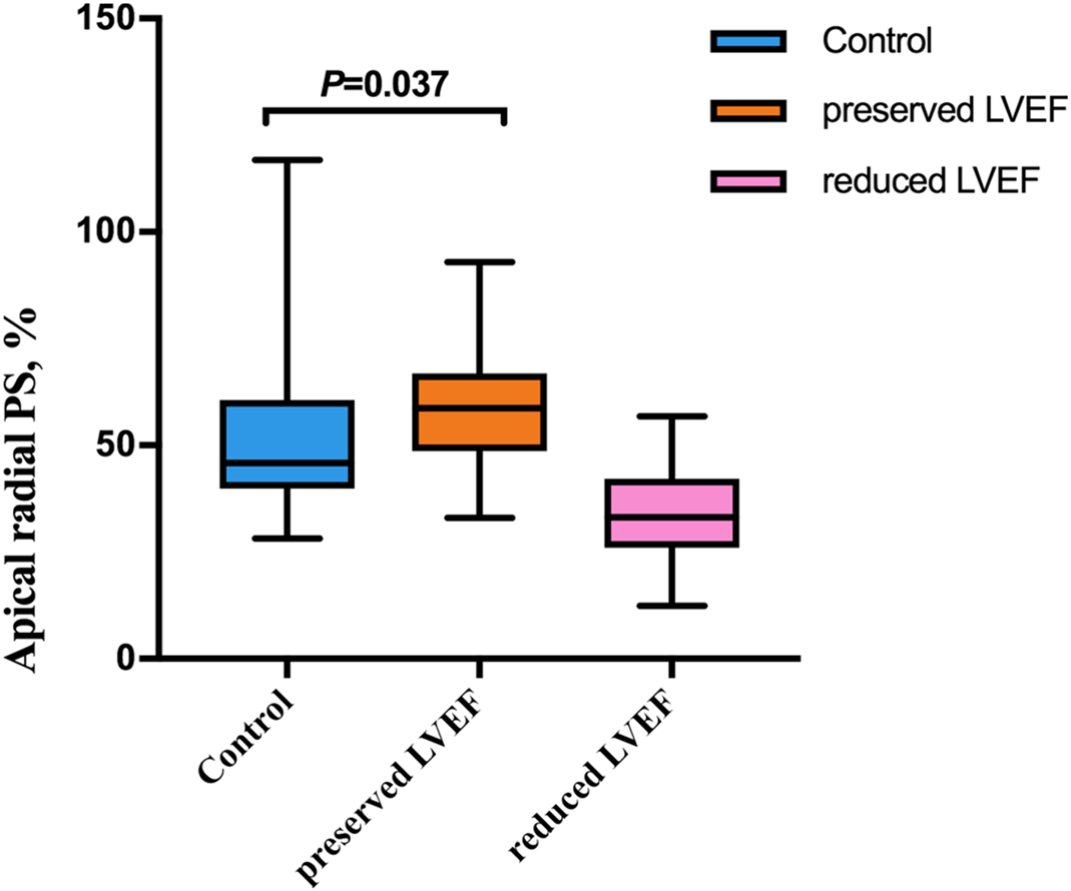
Box plot for the comparison of LV apical radial strain between type 2 diabetes mellitus patients and controls. GraphPad Prism (version 7.0; GraphPad Software).

Regional LV strain parameters (including those of the basal, mid, and apical segments) of the three groups were compared (Supplementary table). Most regional radial, circumferential, and longitudinal LV strain parameters were significantly different between the reduced-LVEF group and control group (all *P* < 0.05). Longitudinal and circumferential PDSR of the basal segment and longitudinal PDSR of the mid segment were lower in the preserved-LVEF group as compared to the control group (all *P* < 0.05). In addition, the radial PS of the apical segment was higher in the preserved-LVEF group as compared to the control group (58.97 ± 13.61% vs. 51.35 ± 18.04%, *P* = 0.037) (Fig. 3).

### Correlations among strain, LVEF, and time-volume curve parameters

**Figure 4:**
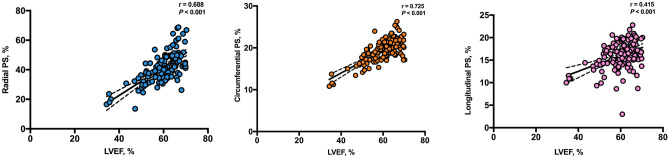
Pearson’s correlation analysis of LVEF with radial peak strain, circumferential peak strain, and longitudinal peak strain. PS, peak strain; LVEF, left ventricular ejection fraction. GraphPad Prism (version 7.0; GraphPad Software).

Significant linear correlations were observed between multiple strain indexes and LVEF (Table 3). LVEF was positively correlated with the radial PS (r = 0.688, *P* < 0.001), circumferential PS (r = 0.725, *P* < 0.001), and longitudinal PS (r = 0.415, *P* < 0.001) (Fig. 4). Weak correlations were found between the LVEF and the time-volume curve parameters (PER and PFR) (Table 3).

**Table 3:** Correlation analysis of LV global strain parameters with the LVEF, PER and PFR.

	LVEF		PER		PFR	
	r	P value	r	P value	r	P value
Radial PS	0.688	0.000	0.197	0.023	0.281	0.001
Circumferential PS	0.725	0.000	0.182	0.036	0.210	0.015
Longitudinal PS	0.415	0.000	0.138	0.114	0.306	0.000
Radial PSSR	0.497	0.000	0.331	0.000	0.346	0.000
Circumferential PSSR	0.398	0.000	0.216	0.013	0.075	0.390
Longitudinal PSSR	0.287	0.001	0.267	0.002	0.204	0.019
Radial PDSR	0.531	0.000	0.183	0.035	0.392	0.000
Circumferential PDSR	0.360	0.000	0.141	0.105	0.402	0.000
Longitudinal PDSR	0.279	0.001	0.153	0.079	0.466	0.000

*PS* peak strain, *PSSR* peak systolic strain rate, *PDSR* peak diastolic strain rate, *LVEF* left ventricular ejection fraction, *PER* peak ejection rate, *PFR* peak filling rate.

### Regression analysis of myocardial strain and LV remodeling

Univariate linear regression analyses showed that HDL, SV, PER, radial PS, longitudinal PS, radial PDSR, circumferential PDSR, and longitudinal PDSR were associated with LVMVR (Table 4). In a multivariate linear regression analysis, model 1 showed that longitudinal PS was independently associated with LVMVR (β = 0.297; *P* = 0.014) and that radial PS was not associated with LVMVR (β =  − 0.036; *P* = 0.755) (Table 4). In model 2, only radial PDSR was independently associated with LVMVR (β =  − 0.242; *P* = 0.042) (Table 4).

**Table 4:** Multivariate linear regression of LVMVR in relation to clinical characteristics, CMR index and global strain.

	LVMVR		LVMVR		LVMVR	
	Univariable		Model 1		Model 2	
	r	P	β	P	β	P
HDL	0.200	0.048	− 0.237	0.007	− 0.218	0.016
SV	0.331	0.001	− 0.185	0.053	− 0.243	0.014
PFR	0.365	0.000	− 0.204	0.036	− 0.155	0.155
Radial PS	0.332	0.001	− 0.036	0.755	–	–
Circumferential PS	0.108	0.290	–	–	–	–
Longitudinal PS	0.421	0.000	0.297	0.014	–	–
Radial PSSR	0.190	0.061	–	–	–	–
Circumferential PSSR	0.111	0.275	–	–	–	–
Longitudinal PSSR	0.195	0.055	–	–	–	–
Radial PDSR	0.387	0.000	–	–	− 0.242	0.042
Circumferential PDSR	0.244	0.015	–	–	0.033	0.796
Longitudinal PDSR	0.313	0.002	–	–	− 0.100	0.415

*HDL* high-density lipoprotein, *SV* stroke volume, *PFR* peak filling rate, *PS* indicates peak strain, *PSSR* peak systolic strain rate, *PDSR* peak diastolic strain rate.

To simplify data management and to select the strain index with the highest value, we performed a preliminary multivariate logistic regression analysis that included only the three determined global PS indexes (Table 5). Of these, only global longitudinal PS was found to be independently associated with LVMVR (β = 0.347; *P* = 0.007). We attempted to determine whether global longitudinal PS would be significantly associated with LVMVR after adjusting for baseline characteristics and whether it would be significantly associated with the CMR indexes that were independently related to the occurrence of LVMVR. In the final multivariate model, global longitudinal PS was found to be associated with LVMVR (Table 6).

**Table 5:** A multivariable logistic regression analysis as predictor of LVMVR.

	LVMVR		
	β	OR (95% IC)	P
Radial PS	− 0.073	2.692	0.101
Circumferential PS	− 0.255	3.368	0.066
Longitudinal PS	0.347	7.325 (1.100–1.819)	0.007

*PS* peak strain.

**Table 6:** A multivariable logistic regression analysis as predictor of LVMVR.

	LVMVR		
	β	Exp (β) (95%IC)	P
**Model 1: longitudinal PS + baseline characteristics**			
Age	0.007	1.007 (0.958–1.059)	0.781
Duration	− 0.024	0.976 0.905–1.053)	0.531
Sex	− 0.097	0.910 (0.328–2.531)	0.857
BMI	0.159	1.172 (0.966–1.424)	0.108
HbA1c	0.226	1.253 (0.649–2.420)	0.502
Hypertension	1.049	2.855 (0.929–8.771)	0.067
Hyperlipidemia	− 0.067	0.935 (0.358–2.441)	0.891
Longitudinal PS	0.366	1.442 (1.174–1.771)	0.000
**Model 2: longitudinal PS + CMR indexes**			
Longitudinal PS	0.385	1.469 (1.193–1.810)	0.000
SV	− 0.034	0.967 (0.917–1.019)	0.210
CO	0.579	1.785 (− 0.965–3.301)	0.065
LVEDD	− 0.044	0.957(0.844–1.085)	0.490
PFR	− 0.008	0.992 (0.984–1.001)	0.072

*BMI* body mass index, *SV* stroke volume, *CO* cardiac output, *LVEDD* left ventricular end-diastolic dimension, *PFR* peak filling rate, *PS* peak strain.

### Analysis of diagnostic performance

**Figure 5:**
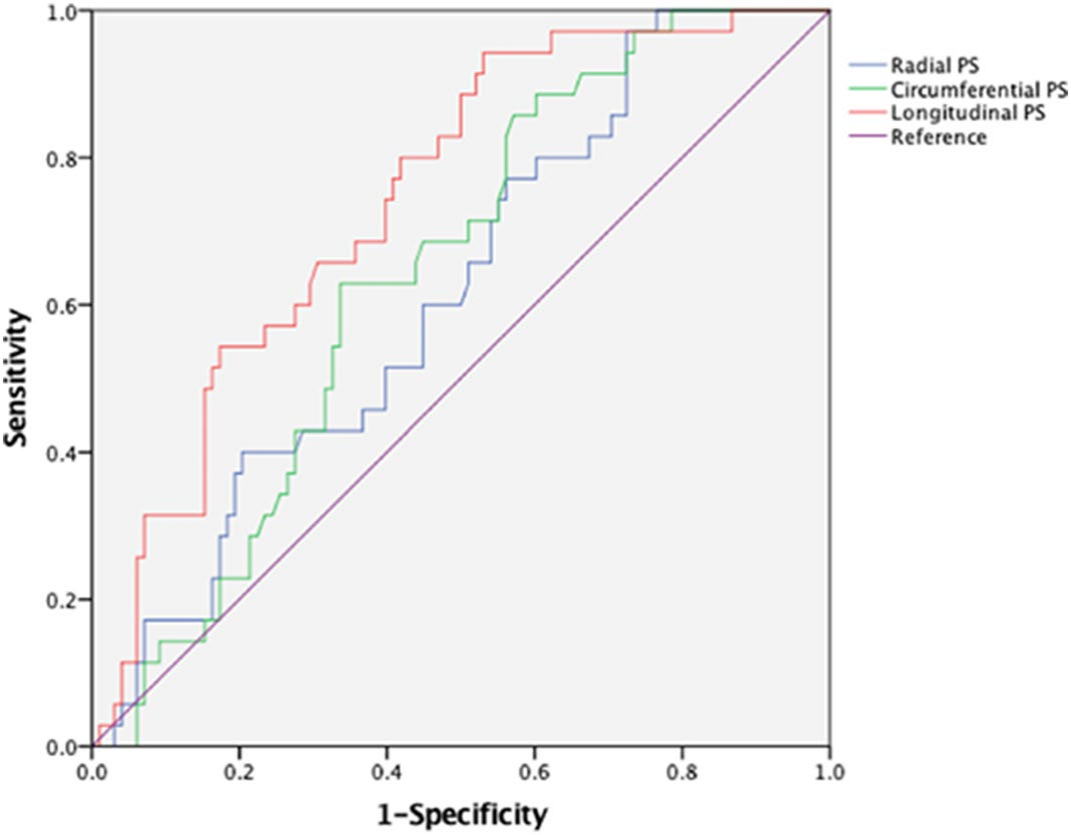
ROC analysis of global radial PS (blue), circumferential PS (green) and longitudinal PS (red) between patients with T2DM and controls. *T2DM* type 2 diabetes mellitus, *PS* peak strain, *ROC* receiver operating characteristic. SPSS (version 24.0; IBM Corp.)

ROC analysis showed the predictive value of LV global radial, longitudinal and circumferential PS for LV dysfunction in T2DM patients (Fig. 5). ROC analysis demonstrated that the area under ROC curve of LV global radial, longitudinal and circumferential PS was 0.616, 0.641 and 0.745.

### Reproducibility of tissue tracking for assessing LV deformation

Reproducibility of tissue-tracking parameters were measured. The inter-observer correlation coefficients (ICC: 0.858–0.964) and intra-observer correlation coefficients (ICC: 0.832–0.920) of radial, circumferential, and longitudinal PS were considered to have good reliability.

## Discussion

Diabetic cardiomyopathy is one of the most common cardiovascular complications, yet no standardized guidelines exist to make the diagnosis and consequently potential cardiovascular complications are often overlooked in the early stages of T2DM. Furthermore, previous studies have mentioned that diabetes has a silent, slow, and early impact on cardiac function^4,22,23^. Although LVEF is traditionally used to monitor cardiac function, it is often preserved or increased in the early stages of diabetes despite the development of global and regional cardiac deformation and dysfunction, indicating that LVEF cannot be reliably used to monitor early subclinical changes in the diabetic heart^24,25^.

The present study adopted traditional CMR cardiac function indexes and time-volume curve parameters and included CMR tissue tracking to assess deteriorated myocardial deformation, including global and regional myocardial strain in the diastolic and systolic periods, in order to comprehensively explore cardiac dysfunction in T2DM, especially those changes occurring in the setting of a preserved LVEF. We demonstrated the value of CMR-derived global longitudinal PS in T2DM patients. Additionally, we found that myocardial strain was closely related to impaired cardiac function and provided imaging information for the early identification of cardiac damage in T2DM patients.

In this study, 72 (73%) out of 98 T2DM patients showed preserved systolic function (LVEF ≥ 55%) and had no clinical cardiac symptoms. We found no significant differences in conventional cardiac function indexes, such as LVEDV, LVESV, LVSV, CO, and LVEDD, between the preserved-LVEF group and the control group. These findings indicate that conventional CMR function parameters might not be of assistance in the early detection of cardiac damage in T2DM patients.

As expected, most CMR tissue-tracking parameters were lower in the reduced-LVEF group as compared to the control group and the preserved-LVEF group, suggesting that T2DM patients with reduced LVEF show both morphological and functional impairments. These findings are consistent with the study by Liu et al. which reported decreased PS in Ebstein’s anomaly with reduced LVEF^26^.

The systolic strain parameters were not significantly lower in the preserved-LVEF group as compared to the control group. However, it is worth noting that the global longitudinal PS and the diastolic strain indicators, such as radial, circumferential, and longitudinal PDSR, were lower in the preserved-LVEF group than the control group. The index PFR for diastolic function in the time-volume curve also decreased, which corresponded to the resulting strain values. In the correlation analysis, there were weak correlations between strain parameters and time-volume curve parameters. A possible reason for the weak correlations is that the principles of evaluation behind these parameters differed. The time-volume curve is based on the change trend of LV volume in a certain period; however, it cannot be used to monitor the systolic and diastolic ability of the myocardium itself. On the other hand, CMR tissue tracking is based on movement of the myocardial voxels for the evaluation of myocardial strain.

Most abnormal global strain parameters occur in the longitudinal direction and are associated with diastolic function, which indicates that strain in the longitudinal direction, and in the diastolic period, becomes impaired in the early stage. These results are consistent with the results of the study by Habek et al. and Nakai et al., which identified cardiac dysfunction in conditions of early diastolic function and late systolic dysfunction in diabetic patients and found that it was associated with heart rate and its variability^27,28^. Previous studies on other heart diseases have shown that most global strain abnormalities first appear in the longitudinal direction, suggesting that longitudinal strain damage may occur early^29–31^. A study about speckle tracking imaging in myocardial infarction also suggested that longitudinal strain damage occurs early^14^. The same abnormal manifestation was observed in this study in T2DM patients. Previous studies have suggested that myocardial interstitial fibrosis and cardiac activity characteristics are closely related to longitudinal strain, which is an important pathophysiological basis of LV remodeling^32,33^ Considering a previous finding that longitudinal PS is better than LVEF for predicting cardiac events^34^, we can assume that the early detection of myocardial strain is of great significance to the clinical prognosis of T2DM patients. ROC curve analysis demonstrated that the performance of global radial, circumferential, and longitudinal PS in detecting T2DM was moderate.

The segmental analysis found that most apical, mid, and basal strain parameters were lower in the reduced-LVEF group as compared with the control group. The trend of change in segmental strain parameters was consistent with that of global LV strain parameters. These results are similar to the findings in the study by Li et al. on cardiac strain in myocardial amyloidosis^35^.

The regional myocardial strain of the basal and mid segments was lower in the preserved-LVEF group as compared with the control group. However, the radial PS of the apical segment was higher in the preserved-LVEF group as compared with the control group. This result may be related to a compensatory increase in the apical radial PS in the early stage, which is consistent with the findings of a previous report^36^.

We found that global strain parameters were positively correlated with LVEF, which indicates that LVEF may decrease with a decrease in global strain. Good correlations were noted between myocardial strain and traditional cardiac function indexes, and high reproducibility and consistency were noted in the diagnosis of cardiac deformation, especially with regards to the early evaluation of subclinical diastolic dysfunction in T2DM.

Previous studies have shown that diabetes is correlated with LV remodeling, which may lead to systolic or diastolic dysfunction^5^. Our results indicate that patients with diabetes have an increased LV remodeling index (LVMVR) when compared with the value in normal individuals. In our study, we also explore the relationship between strain parameters and the LV remodeling index. Global longitudinal PS, radial PDSR, circumferential PDSR, and longitudinal PDSR were the only strain parameters independently related to LVMVR. A previous study has suggested that longitudinal PS is better than LVEF for predicting cardiac events31. Based on these findings, we only included global longitudinal PS in subsequent multivariate logistic regression analyses.

In the multivariate logistic regression analyses, we found that global longitudinal PS (derived from strain parameters) was associated with the occurrence of LVMVR. After comprehensive adjustments for baseline and traditional CMR indexes, only global longitudinal PS was associated with LV remodeling.

### Limitations

The present study has some limitations. First, this study did not provide long-term follow-up data. Second, this study only evaluated LV myocardial strain; right ventricular strain characteristics are currently being considered in a follow-up study. Finally, CMR tissue tracking is a technology that involves increased automatization and robustness of the qualitative analysis of cardiac LV strain dysfunction. Although high reproducibility was achieved in our study, the accuracy of this approach needs to be further validated owing to the lack of a reference standard.

## Conclusion

The assessment of strain parameters obtained via CMR tissue tracking, allows for the evaluation of early cardiac deformation in T2DM patients. In addition, global longitudinal PS can complement LVEF in the assessment of cardiac function.

## Supplementary information

Supplementary Information.
