# Supplementary material for: Assessment of left ventricular deformation in patients with type 2 diabetes mellitus by cardiac magnetic resonance tissue tracking
Source: Sci Rep. 2020 Aug 4;10:13126. doi: 10.1038/s41598-020-69977-x (PMC7403307; doi:10.1038/s41598-020-69977-x)
Supplement: Supplementary file 1 — Supplementary Information. [file 41598_2020_69977_MOESM1_ESM.pdf]

Assessment of left ventricular deformation in patients with type 2 diabetes mellitus by cardiac magnetic resonance tissue tracking

Lin-jun Xie[1]†, MS; Zhi-hui Dong[2]†, MD; Zhi-gang Yang[3*], MD; Ming-yan Deng[4], MS; Yue Gao[3], MS; Li Jiang[3], MD; Bi-yue Hu[3], MS; Xi Liu[3], MD; Yan Ren[4], MD; Chun-chao Xia[3], MS; Zhen-lin Li[3], MS; Hua-peng Zhang[5], MS; Xiao-yue Zhou[5], MD; Ying-kun Guo[1*], MD

1. Department of Radiology; Key Laboratory of Obstetric & Gynecologic and Pediatric Diseases and Birth Defects of Ministry of Education; West China Second University Hospital, Sichuan University; 20# South Renmin Road, Chengdu, Sichuan 610041, China.
2. Department of Radiology, Luoyang Central Hospital Affiliated to Zhengzhou University; 288# Zhongzhou Middle Road, Luoyang, Henan 471009, China.
3. Department of Radiology, West China Hospital, Sichuan University, 37# Guo Xue Xiang, Chengdu, Sichuan 610041, China.
4. Department of Endocrinology and Metabolism, West China Hospital, Sichuan University, 37# Guo Xue Xiang, Chengdu, Sichuan 610041, China.
5. MR Collaboration, Siemens Healthineers Ltd., Shanghai, China.

†. Lin-jun Xie and Zhi-hui Dong contributed equally to this work.
*. Ying-kun Guo and Zhi-gang Yang contributed equally to this work and should be considered co-corresponding authors.
*Corresponding author: gykpanda@163.com

Left ventricle segmental deformation difference between normal patients, preserved and reduced LVEF T2DM patients

	Basal			Mid			Apical		
	Normal n=35	Patients with preserved LVEF n=72	Patients with reduced LVEF n=26	Normal n=35	Patients with preserved LVEF n=72	Patients with reduced LVEF n=26	Normal n=35	Patients with preserved LVEF n=72	Patients with reduced LVEF n=26
**PS(%)**									
Radial	51.32±11.47	49.23±12.30	38.27±9.98*§	37.28±7.38	36.66±9.57	23.40±7.72*§	51.35±18.04	58.97±13.61	33.77±11.57*§
Circumferential	-17.90±2.01	-17.67±2.41	14.26±3.02*§	-22.81±1.72	-22.85±2.71	-18.10±2.85*§	-21.13±2.28	-21.61±3.18	-17.26±3.31*§
Longitudinal	-13.07±3.75	-10.69±5.00*	10.59±3.62*	-21.62±3.03	-20.31±4.93	-17.04±4.07*§	-19.68±2.18	-19.62±3.53	-15.91±3.34*§
**PSSR (1/S)**									
Radial	3.55±1.31	3.40±1.74	2.74±1.39*	2.16±0.91	2.01±1.57	1.31±0.89*§	3.73±1.77	3.44±2.74	2.11±0.75*§
Circumferential	-0.92±0.16	-0.90±0.34	-0.73±0.15*§	-1.25±0.26	-1.18±0.37	-0.87±0.47*§	-1.32±0.26	-1.22±0.59	-0.91±0.52*§
Longitudinal	-0.73±0.33	-0.48±0.69*	-0.57±0.37	-1.17±0.27	-1.06±0.38	-0.93±0.21*	-1.28±0.41	-1.18±0.58	-0.98±0.75*
**PDSR (1/S)**									
Radial	-3.95±3.08	-4.05±1.94	-3.08±1.13§	-3.00±1.32	-2.80±1.00*	-1.76±0.67*§	-4.25±1.79	-4.02±2.41	-1.91±2.10*§
Circumferential	1.20±0.26	1.05±0.26*	0.89±0.27*§	1.52±0.34	1.38±0.30*	1.21±0.32*§	1.42±0.30	1.40±0.38	1.13±0.33*§
Longitudinal	0.98±0.46	0.77±0.58*	0.75±0.32	1.48±0.35	1.23±0.38*	1.10±0.36*	1.39±0.37	1.34±0.49	1.07±0.62*

PS, peak strain; PSSR, peak systolic strain rate; PDSR, peak diastolic strain rate.
* $P < 0.05$ versus normal group; § $P < 0.05$ versus T2DM with preserved LVEF
